# Supplementary material for: Lysosomal integral membrane protein-2 as a phospholipid receptor revealed by biophysical and cellular studies
Source: Nat Commun. 2017 Dec 4;8:1908. doi: 10.1038/s41467-017-02044-8 (PMC5712522; doi:10.1038/s41467-017-02044-8)
Supplement: Supplementary file 1 — Supplementary Information [file 41467_2017_2044_MOESM1_ESM.pdf]

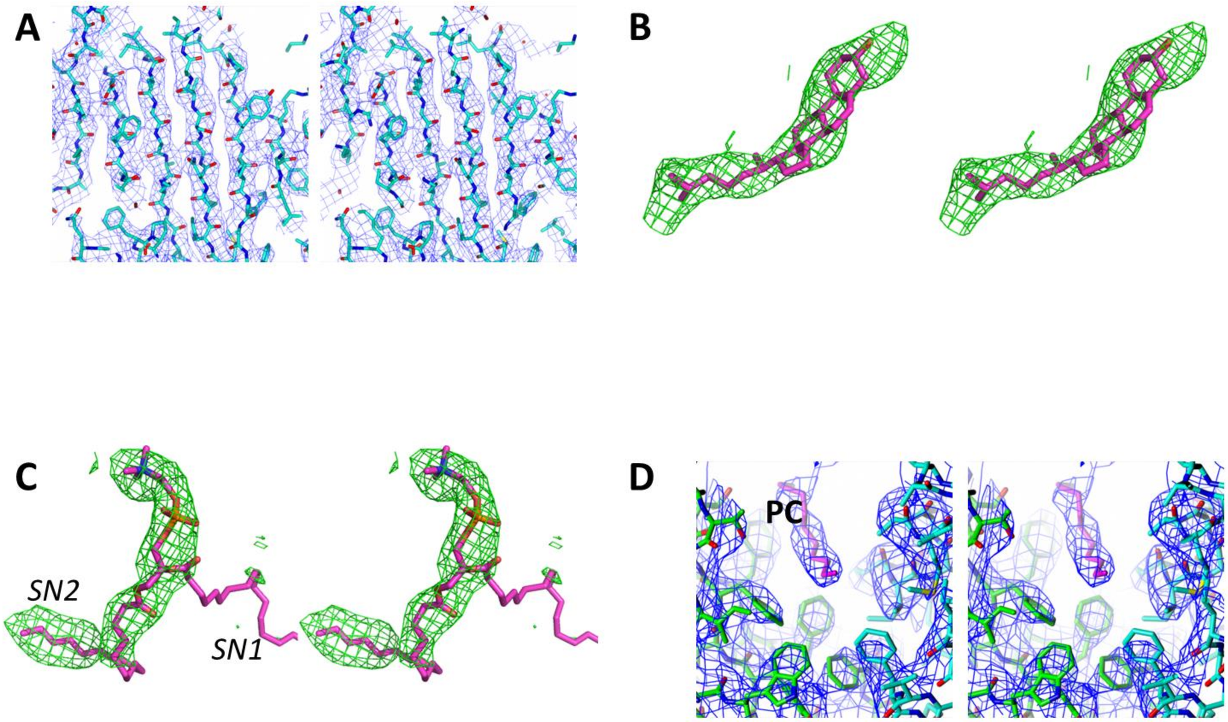

Supplementary Figure 1. Examples of electron density illustrate quality of model fitting. A. Stereo view of a representative of the final 2Fo-Fc electron density (blue mesh, contoured at 1.0 σ) embedded with the final refined lipids bound LIMP-2 dimer model (sticks, carbon atoms colored in cyan, nitrogen blue and oxygen red). B. Stereo view of the initial Fo-Fc omit map (green mesh, contoured at 3σ) into which a cholesterol model was built. C. The initial Fo-Fc map for one of the PC molecules at the hydrophobic tunnel. Acyl chains of the PC are also labelled. D. Electron density around one of the hydrophobic clefts with a fragment of PC built.

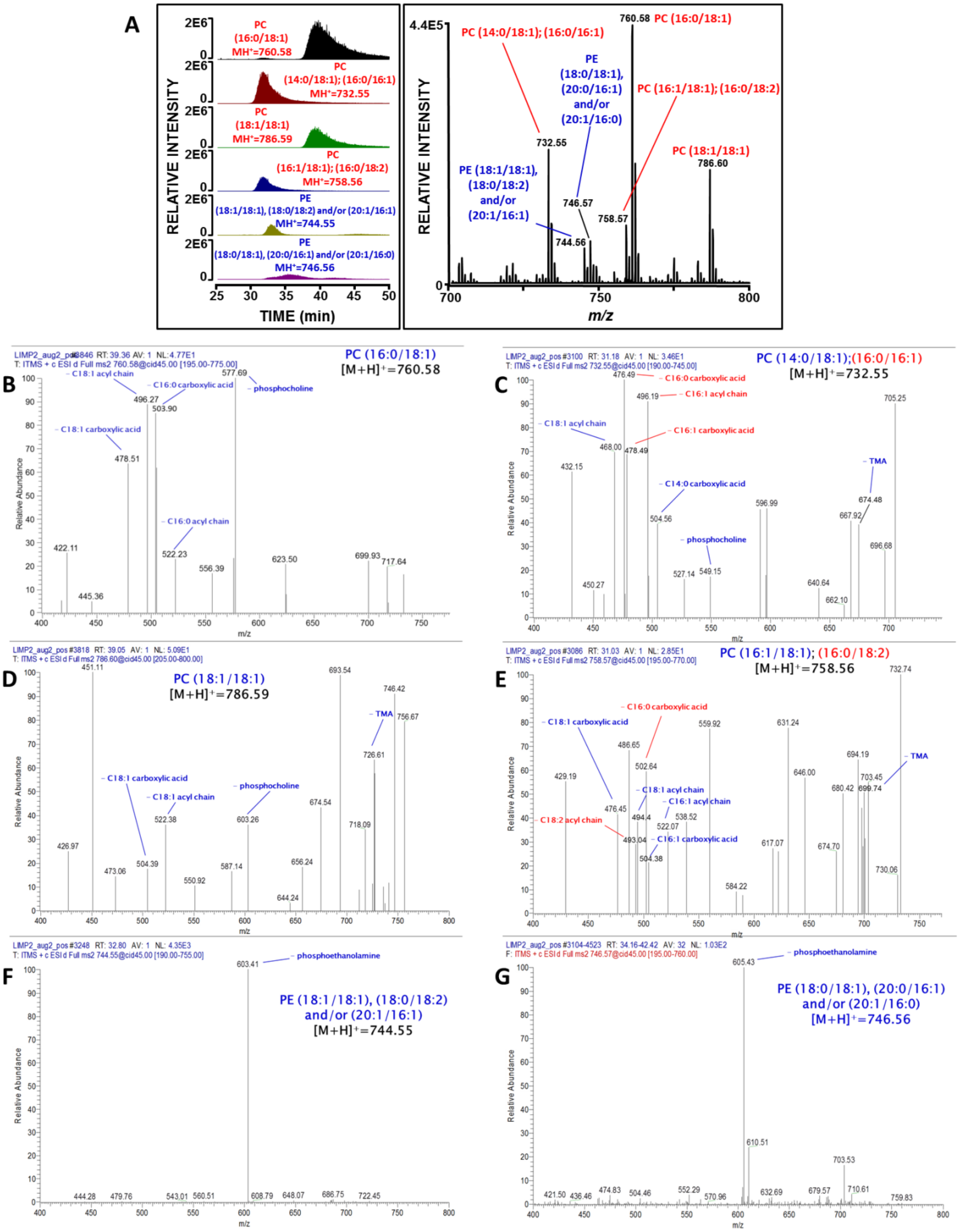

A
RELATIVE INTENSITY
TIME (min)
PC (16:0/18:1) MH+=760.58
PC (14:0/18:1); (16:0/16:1) MH+=732.55
PC (18:1/18:1) MH+=786.59
PC (16:1/18:1); (16:0/18:2) MH+=758.56
PE (18:1/18:1), (18:0/18:2) and/or (20:1/16:1) MH+=744.55
PE (18:0/18:1), (20:0/16:1) and/or (20:1/16:0) MH+=746.56
m/z
B
PC (16:0/18:1) [M+H]+=760.58
C
PC (14:0/18:1);(16:0/16:1) [M+H]+=732.55
D
PC (18:1/18:1) [M+H]+=786.59
E
PC (16:1/18:1); (16:0/18:2) [M+H]+=758.56
F
PE (18:1/18:1), (18:0/18:2) and/or (20:1/16:1) [M+H]+=744.55
G
PE (18:0/18:1), (20:0/16:1) and/or (20:1/16:0) [M+H]+=746.56
Relative Abundance
phosphocholine
phosphoethanolamine
TMA

Supplementary Figure 2. Identification of phospholipids species in purified LIMP-2 samples using MS. A. MS extract ion plot and spectrum of PC and PE in positive-ion mode present in purified LIMP-2. Cholesterol was not detectable using this experiment. PS could be detected in standards under conditions used, but not detected in our LIMP-2 samples. B-G. Identifications of phospholipid species in LIMP-2 samples by positive electrospray ionization ion electron spray MS/MS analysis. B. Positive ion ESI MS/MS-spectra of the $[M+H]^+$ =760.58 of PC (16:0/18:1). C. The $[M+H]^+$ =732.55 of PC (C14:0/C18:1) ; (16:0/16:1) species. D. $[M+H]^+$ =786.59 of PC (18:1/18:1). E. $[M+H]^+$ =758.56 of PC (16:1/18:1); (16:0/18:2). F. $[M+H]^+$ =744.55 of PE 18:1/18:1), (18:0/18:2) and/or (20:1/16:1). G. $[M+H]^+$ =746.56 of PE (18:0/18:1), (20:0/16:1) and/or (20:1/16:0).

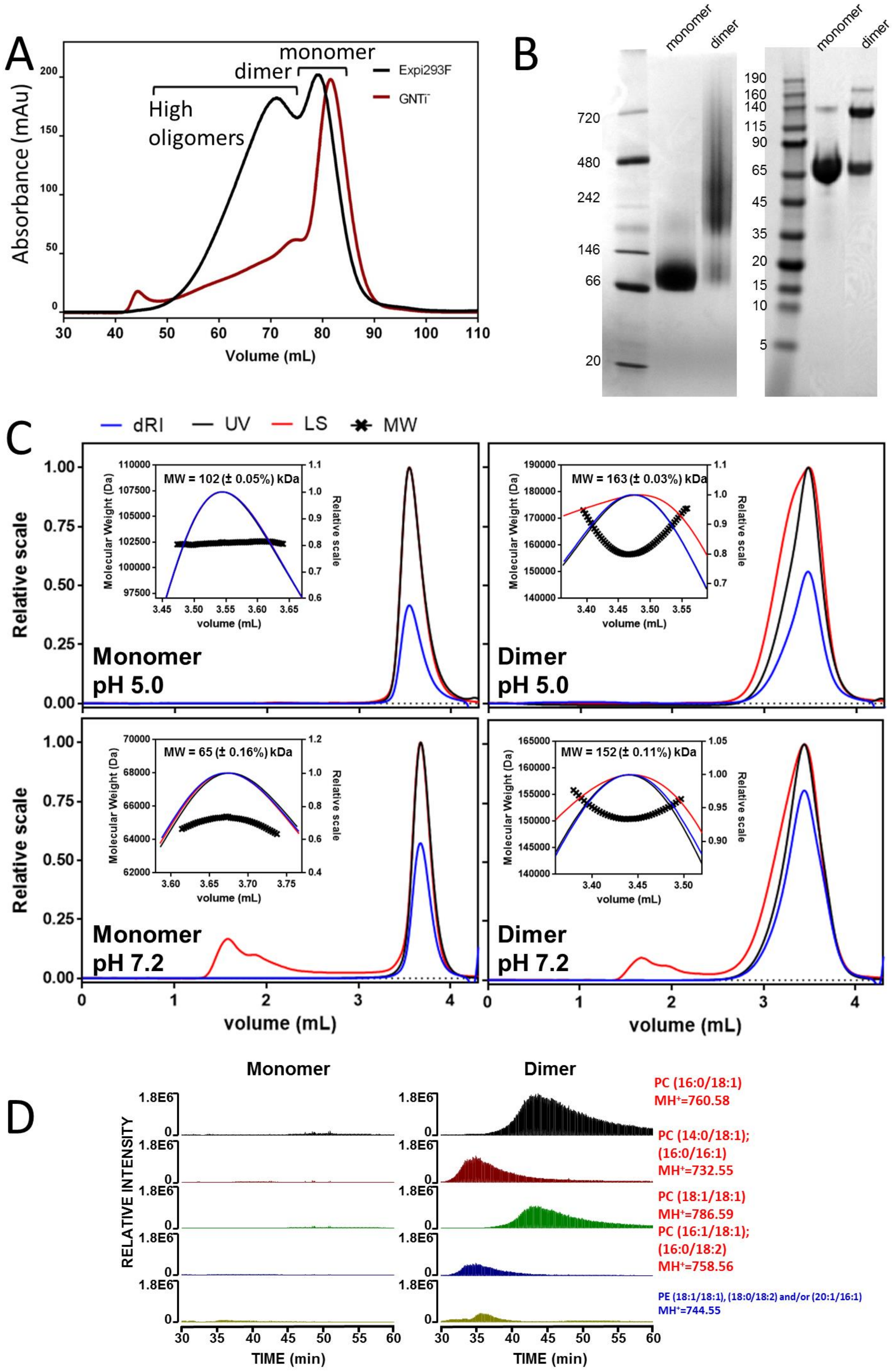

A
B
C
D
High oligomers
dimer
monomer
Expi293F
GNTi⁻
Absorbance (mAu)
Volume (mL)
monomer
dimer
dRI
UV
LS
MW
MW = 102 (± 0.05%) kDa
MW = 163 (± 0.03%) kDa
MW = 65 (± 0.16%) kDa
MW = 152 (± 0.11%) kDa
Relative scale
Molecular Weight (Da)
volume (mL)
Monomer pH 5.0
Dimer pH 5.0
Monomer pH 7.2
Dimer pH 7.2
Monomer
Dimer
RELATIVE INTENSITY
TIME (min)
PC (16:0/18:1) MH⁺=760.58
PC (14:0/18:1); (16:0/16:1) MH⁺=732.55
PC (18:1/18:1) MH⁺=786.59
PC (16:1/18:1); (16:0/18:2) MH⁺=758.56
PE (18:1/18:1), (18:0/18:2) and/or (20:1/16:1) MH⁺=744.55

Supplementary Figure 3. Ligand bound LIMP-2 luminal domain exists as dimer in solution. A. LIMP-2 luminal domain expressed in the wild type (WT) mammalian cells (HEK293F) elutes mainly as a mixture of monomer and dimer on size exclusion chromatography (SEC; Superdex 200, 16-60), with a tail of higher oligomers. Consistent with the observed dimer crystal structure, the same LIMP-2 construct expressed in glycosylation deficient cells (HEK293-GnTi-) demonstrates a large shift in SEC profile toward monomer and decrease in dimer peak height by ~3/4. B. Molecular weights of LIMP-2 monomer and dimer determined on 4-16% NativePAGE Bis-Tris Gel (left) and 4-12% Bis-Tris SDS Gel (right, with reducing agent). C. Molecular weights of LIMP-2 monomer and dimer (Expi293F cells) in solution determined by SEC-MALS. For B and C, pooled monomer and dimer fractions from anion exchange chromatography, following the initial separation on SEC were used. dRI, differential refractive index; UV, UV absorbance 280 nm; LS, light scattering; MW, molecular weight. Top and bottom panels are at pH=7.2 and 5.0, respectively. Left and right panels are LIMP-2 monomer and dimer, respectively. At pH 7.2, the molecular weight of LIMP-2 monomer and dimer are 65.0(±0.16%) and 152(±0.11%) KD, respectively, corresponding to those of the fully glycosylated species. At pH 5.0, the molecular weights of LIMP-2 samples that behave normally as monomer and dimer at pH 7.2 increase to 102.0 (±0.05%) and 163 (±0.03%) kDa, respectively, indicating that LIMP-2 has a strong propensity to dimerize at acidic pH. D. Example of LC/MS spectra to show that the amounts of endogenous phospholipids were greatly reduced in the LIMP-2 monomer samples (left), while they were always detected in the wild type LIMP-2 dimer (right). The amounts of protein samples were used as internal standards to normalize lipids heights. The lipid contents in the monomer were 16±8% (N=2) of those in the dimer judged by the areas of MH^+=760.58, the most abundant lipid species. The monomer and dimer samples were from the same batch of protein expressed, carefully separated on the SEC, and then went through the same ion exchange procedures.

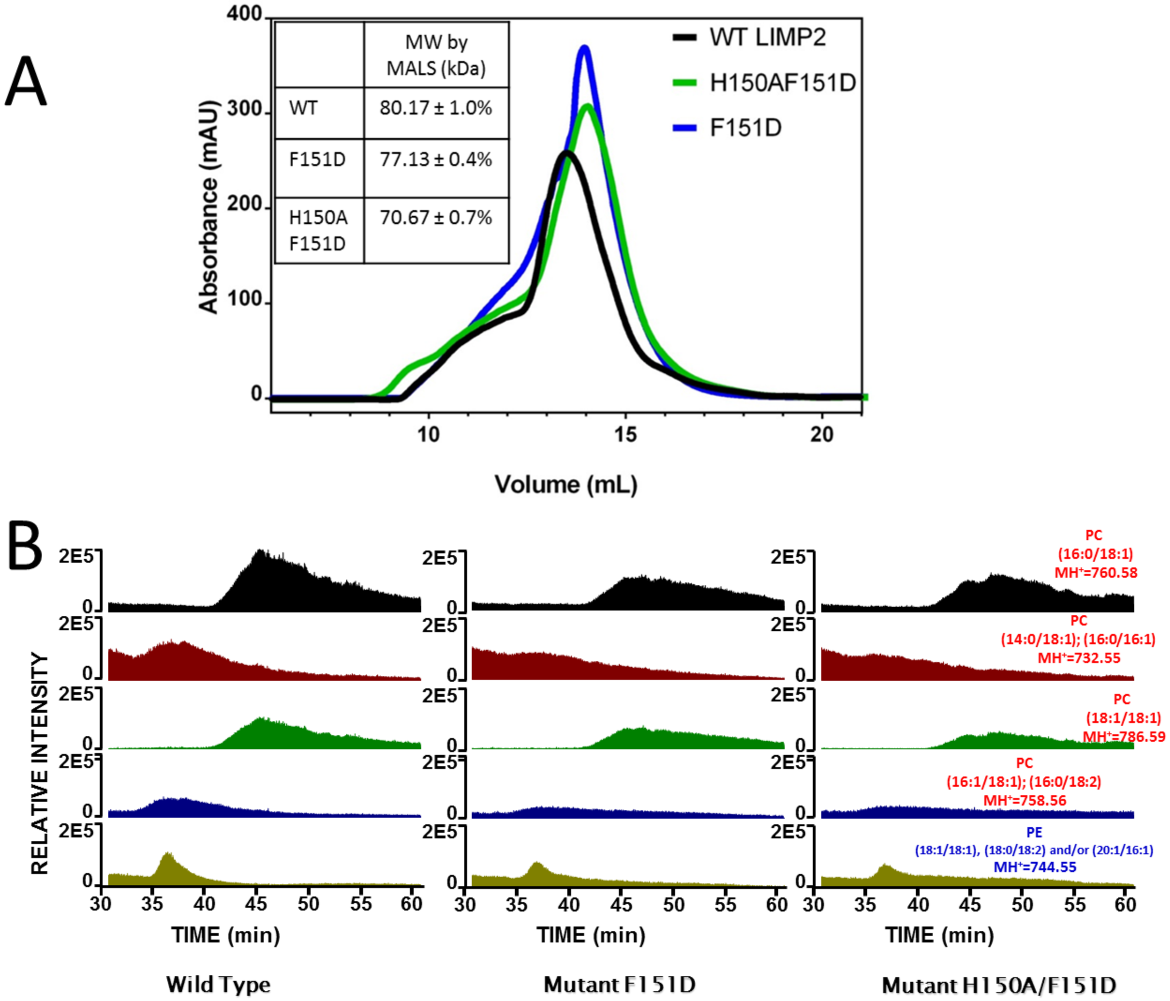

Supplementary Figure 4. Dimer interface mutations decrease the amounts of dimer in LIMP-2. A, the size exclusion profiles (Superdex 200 Increase, 10/300) of the wild type, the dimerization defective mutants, F151D and H150AF151D, of LIMP-2 (Expi293F cells). Insets are molecular weights of the main size exclusion peaks of these proteins determined by MALSB. B. In consistent with that lipids binding favors LIMP-2 dimer formation, the amounts of bound endogenous phospholipids detected by LC/MS decrease in the main SEC peak fractions of F151D and H150A/F151D mutants, relative to those in the wild type LIMP-2. (by 40±1% and 36.5±1.5%, N=2, quantified by areas of PC $[M+H]^+$ 760 species, respectively). Same amounts of protein samples were applied in LC/MS analysis.

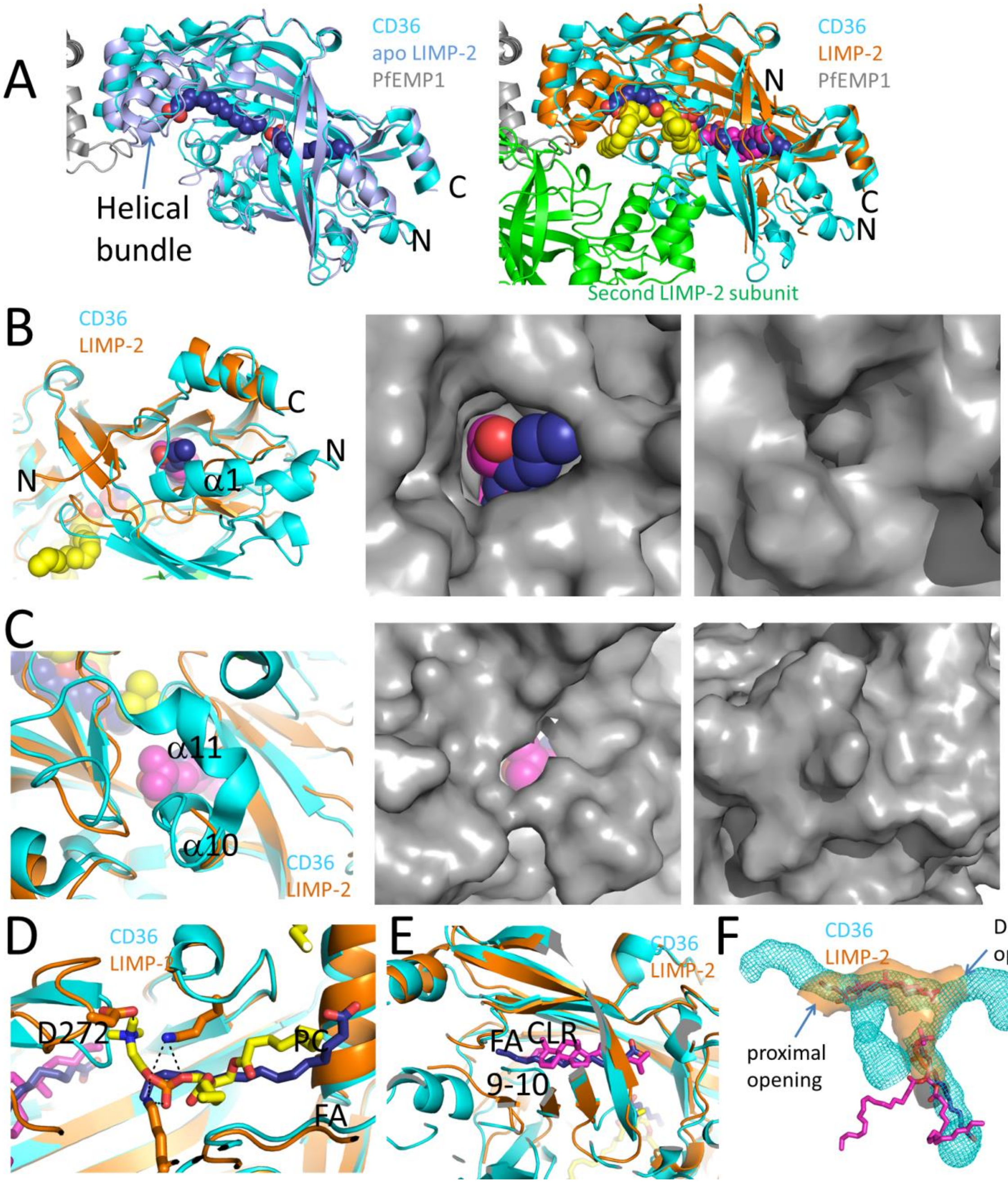

Supplementary Figure 5. The structure of CD36 monomer is very different from subunits in LIMP-2 dimer. A. The structure of fatty acids (FA) bound CD36 monomer in complex with PfEMP1 (PDB code 5JDG, CD36 and portions of PfEMP1 are shown as cyan and gray ribbons, respectively; bound fatty acids are shown in spheres with carbon atoms colored dark blue) is highly similar to that of apo LIMP-2 monomer (light blue ribbons, PDB 4Q4F) (left) but very different from subunits in the LIMP-2 dimer (one subunit in orange ribbons; bound PC and CLR molecules with carbon atoms colored yellow and magenta, respectively. Portion of the second

LIMP-2 subunit is shown in green ribbons) (right). N- and C-termini are indicated. B and C, the hydrophobic tunnel in CD36 monomer is unlikely to facilitate the uptake of fatty acid due to the blockage of the openings. Displacement of α1 helix due to dimerization unblocks the exit of the fatty acid/cholesterol site (B, left) in the LIMP-2 dimer, making it open to the membrane proximal face of the LIMP-2 dimer (B, middle, protein in surface presentation). This exit is largely blocked by α1 helix in the CD36 monomer structure (B, right). Displacement of α10 and α11 helices by the opposite subunit in the LIMP-2 dimer (C, left) creates a second opening on the distal face of the LIMP-2 dimer for the fatty acid/cholesterol site (C, middle). This second opening is also missing in the CD36 monomer structure (C, right). D and E, Ligand binding site in CD36 and subunit of LIMP-2 dimer. The phospholipid in LIMP-2 and the fatty acid (FA) closer to the entrance in CD36 differ on account of the PC amine headgroup is able to anchor to D272 and displace the peptide it locates. The cholesterol and fatty acid deep in the lipid binding tunnel occupy the same space, but the cholesterol is bulkier and able to displace peptide 9-10, but FA is not. F. Superimposition of the hydrophobic tunnel in cholesterol and PC bound LIMP-2 subunit (orange solid surface) with that of the FA bound CD36 (cyan mesh surface). The distal and proximal openings of LIMP-2 are labeled.

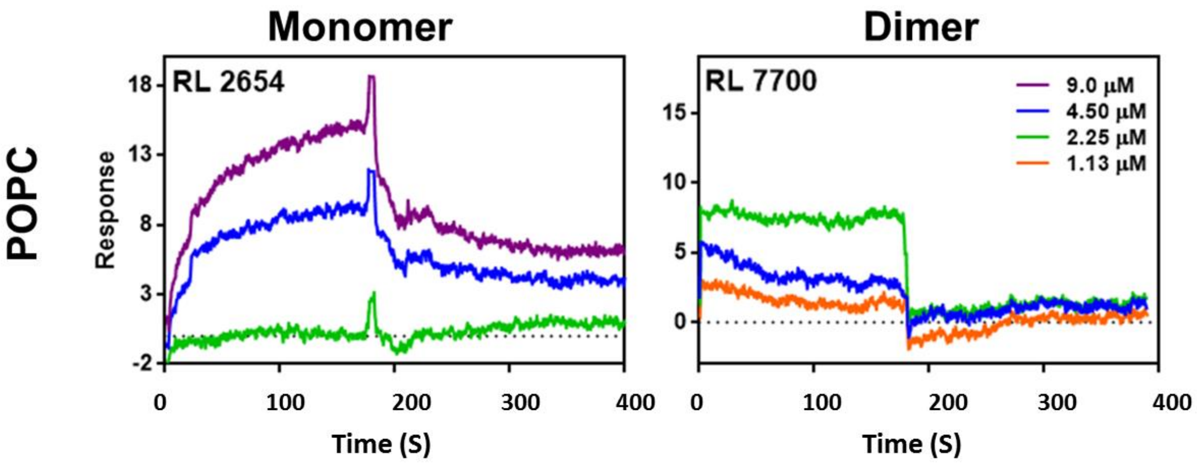

Supplementary Figure 6. SPR sensorgrams of LIMP-2 binding to immobilized POPC liposomes at variable immobilization levels. LIMP-2 monomer (left) binding to POPC liposomes required high lipid levels and high protein concentrations, indicating that it has to dimerize on lipids for binding. The liposome immobilization levels (RL) and protein concentrations were indicated. LIMP-2 dimer (right) did not show any binding kinetics to POPC liposomes under any conditions tested. Representative sensorgrams are from duplicated experiments.

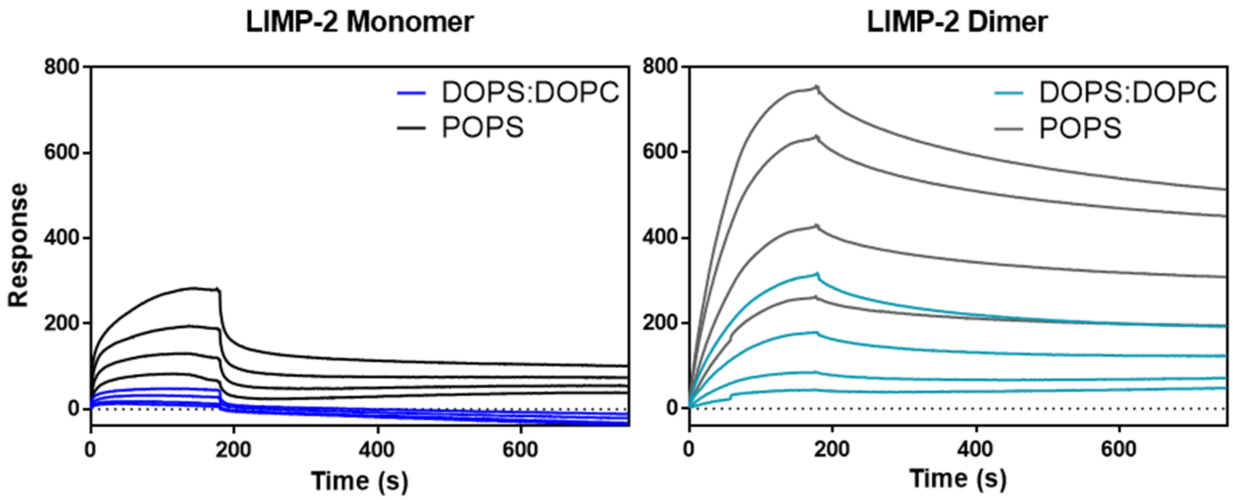

Supplementary Figure 7. Example of SPR sensorgrams of LIMP-2 monomer and dimer binding to DOPS:DOPC and POPS liposomes at pH 5.0. SPR channel in which POPC was immobilized at similar level as reference for subtraction (RU: 4.7K, 6.8k and 3.2K, respectively). At this pH, LIMP-2 bound blank L1 chip supporting matrix, and negative binding responses of LIMP-2 were observed relative to the matrix when POPC was immobilized. Both LIMP-2 monomer and dimer had strong positive binding responses when POPS and DOPS:DOPC liposomes were immobilized. Kinetic fitting was not attempted. Concentrations used: 0.281, 0.563, 1.13 and 2.25 μM. Notice that LIMP-2 dimer has much stronger binding responses than LIMP-2 monomer, and responses increase with increasing contents of PS in the liposome.

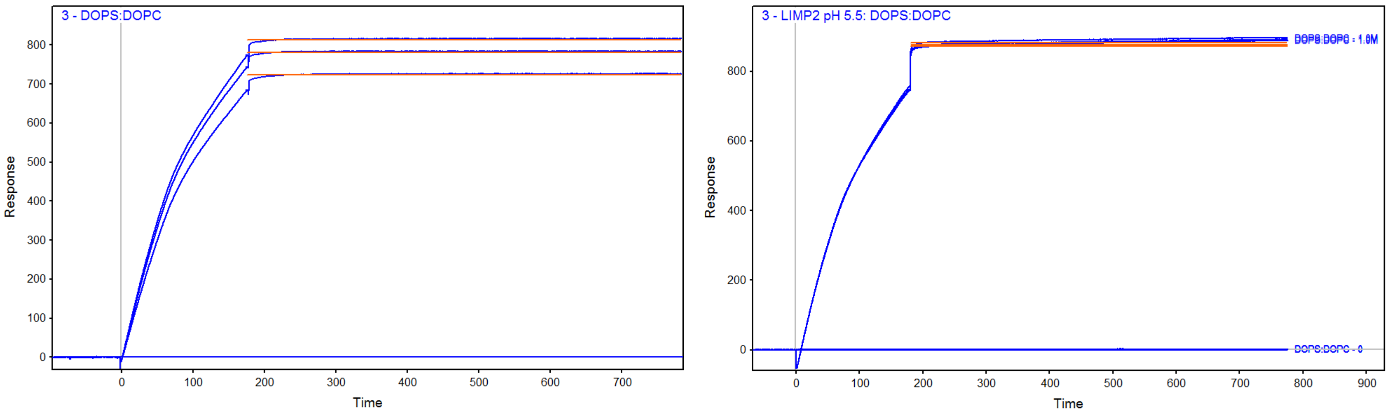

Supplementary Figure 8. Examples of SPR sensorgrams of the pseudo irreversible binding behaviors of liposome to immobilized LIMP-2. DOPC:DOPS (3:2) was used in the mobile phase and LIMP-2 was immobilized via biotin tag to neutroavidin surface. Left, pH=7.5, right, pH.5.5. Measurement was triplicated at 1 μM liposome concentration.

A

B

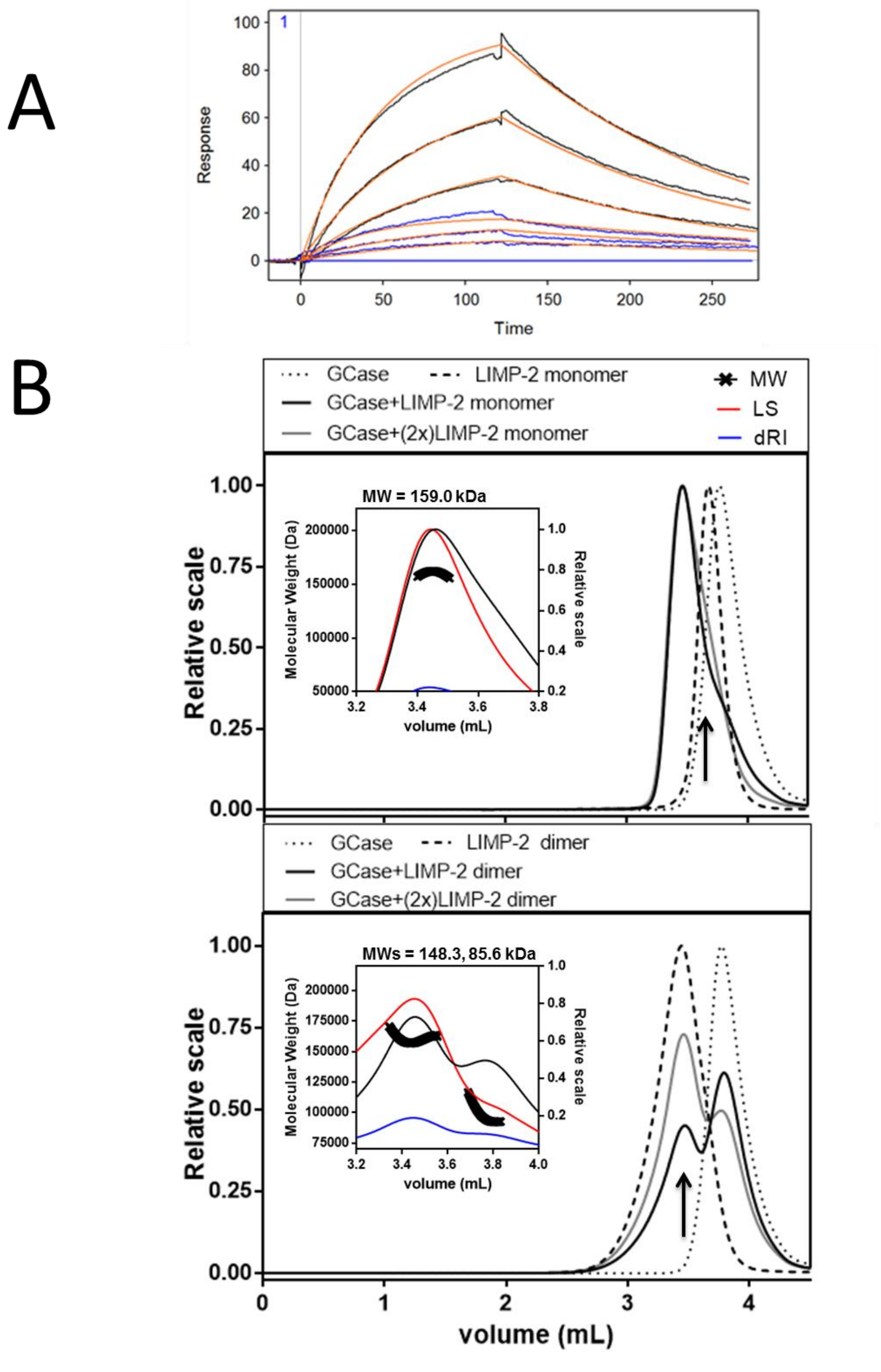

Response
Time
GCase
LIMP-2 monomer
GCase+LIMP-2 monomer
GCase+(2x)LIMP-2 monomer
MW
LS
dRI
MW = 159.0 kDa
Molecular Weight (Da)
Relative scale
volume (mL)
GCase
LIMP-2 dimer
GCase+LIMP-2 dimer
GCase+(2x)LIMP-2 dimer
MWs = 148.3, 85.6 kDa
volume (mL)

Supplementary Figure 9. LIMP-2 monomer binds GCase but dimer binding is diminished. A. Fitting curves (orange) and SPR sensorgrams of LIMP-2 monomer (black) and dimer (blue) binding to GCase immobilized via biotin tag. The kinetic fitting parameters for the monomer are k_a=2.9 (±0.5)x10^{-4} $M^{-1}S^{-1}$, k_d=5.7(±1.0)x10-3 S^{-1}, K_D= 212(±80) nM, and R_{max}=147.2 (from all concentrations of duplicated experiments). The responses of LIMP-2 dimer sample are only fractions of those of monomer at the same concentrations. The small responses of the dimer can be best fitted with bindings only from a fraction of monomer species that exists in the dimer sample, resulting in a very similar K_D but ~1/7 of R_{max} of the monomer. B. SEC-MALS experiments demostrating that LIMP-2 monomer forms a 1:1 complex with GCase in soultion, but no complex containing a LIMP-2 dimer can be detected. Notice that protein peak corresponding to free GCase disappeared when mixed with LIMP-2 monomer, but remained when mixed with LIMP-2 dimer. Inserts show the molecular weights determined by SEC-MALS. Arrow in the top panel indicates the position of the LIMP-2 monomer, and in the bottom, the position of the dimer.

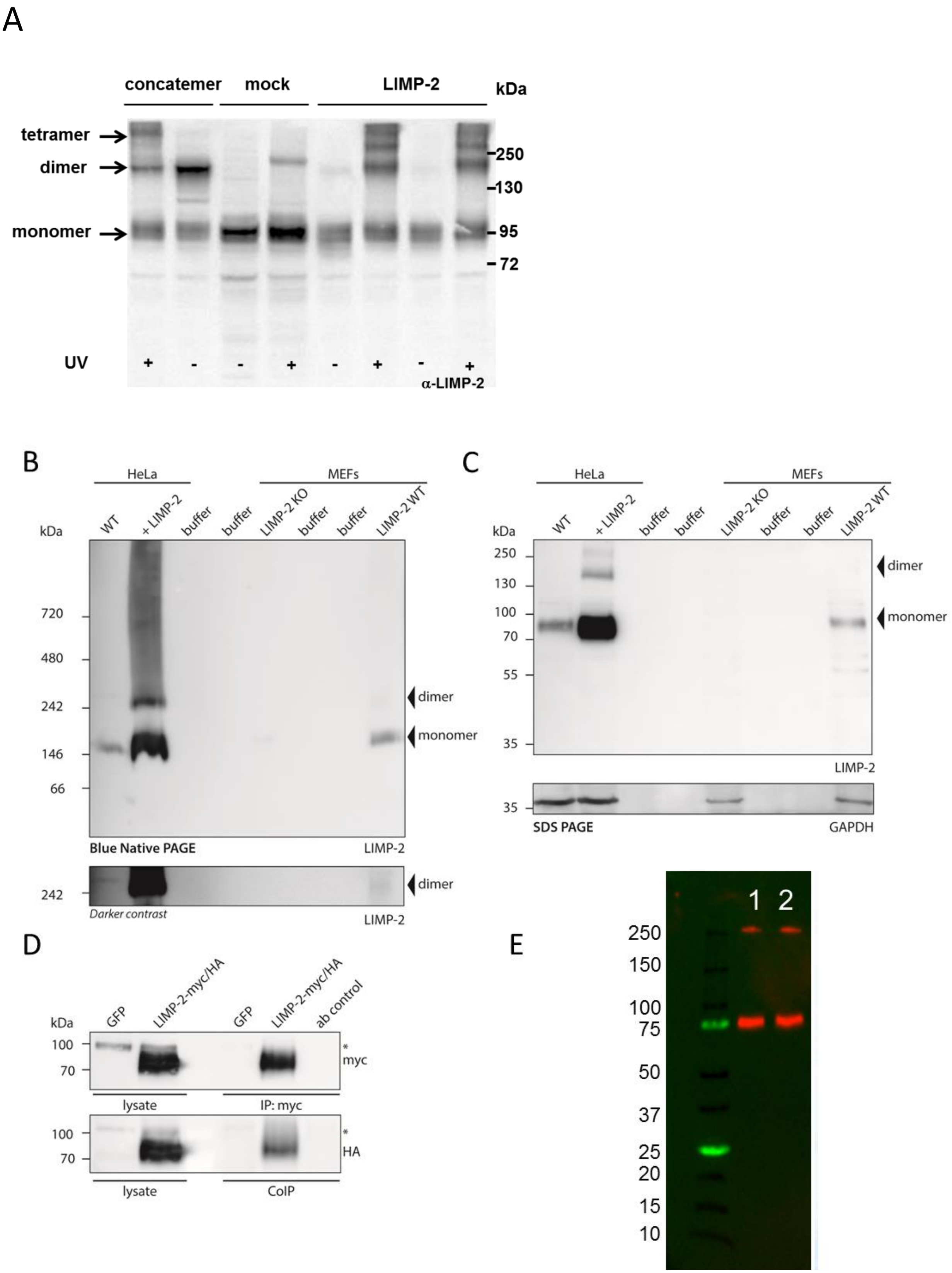
A
concatemer
mock
LIMP-2
kDa
tetramer
dimer
monomer
250
130
95
72
UV
+
-
-
+
-
+
-
+
α-LIMP-2
B
HeLa
MEFs
WT
+ LIMP-2
buffer
buffer
LIMP-2 KO
buffer
buffer
LIMP-2 WT
kDa
720
480
242
146
66
dimer
monomer
Blue Native PAGE
LIMP-2
242
dimer
Darker contrast
LIMP-2
C
HeLa
MEFs
WT
+ LIMP-2
buffer
buffer
LIMP-2 KO
buffer
buffer
LIMP-2 WT
kDa
250
130
100
70
55
35
dimer
monomer
LIMP-2
35
SDS PAGE
GAPDH
D
kDa
GFP
LIMP-2-myc/HA
GFP
LIMP-2-myc/HA
ab control
100
70
*
myc
lysate
IP: myc
100
70
*
HA
lysate
CoIP
E
1
2
250
150
100
75
50
37
25
20
15
10

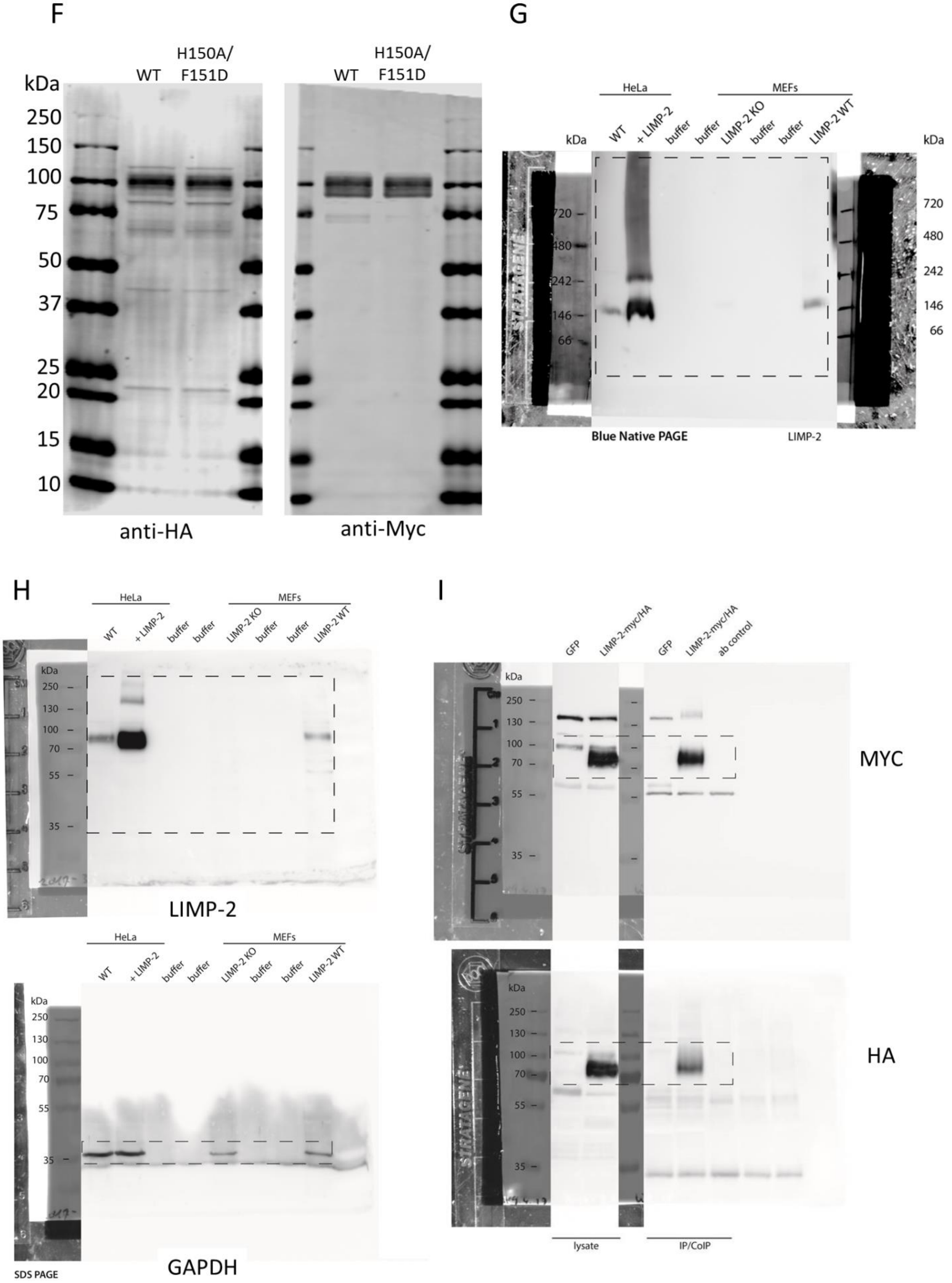
F
kDa
WT
H150A/
F151D
250
150
100
75
50
37
25
20
15
10
anti-HA
anti-Myc
G
HeLa
MEFs
kDa
WT
+ LIMP-2
buffer
buffer
LIMP-2 KO
buffer
buffer
LIMP-2 WT
720
480
242
146
66
Blue Native PAGE
LIMP-2
H
LIMP-2
GAPDH
SDS PAGE
I
GFP
LIMP-2-myc/HA
GFP
LIMP-2-myc/HA
ab control
MYC
HA
lysate
IP/CoIP

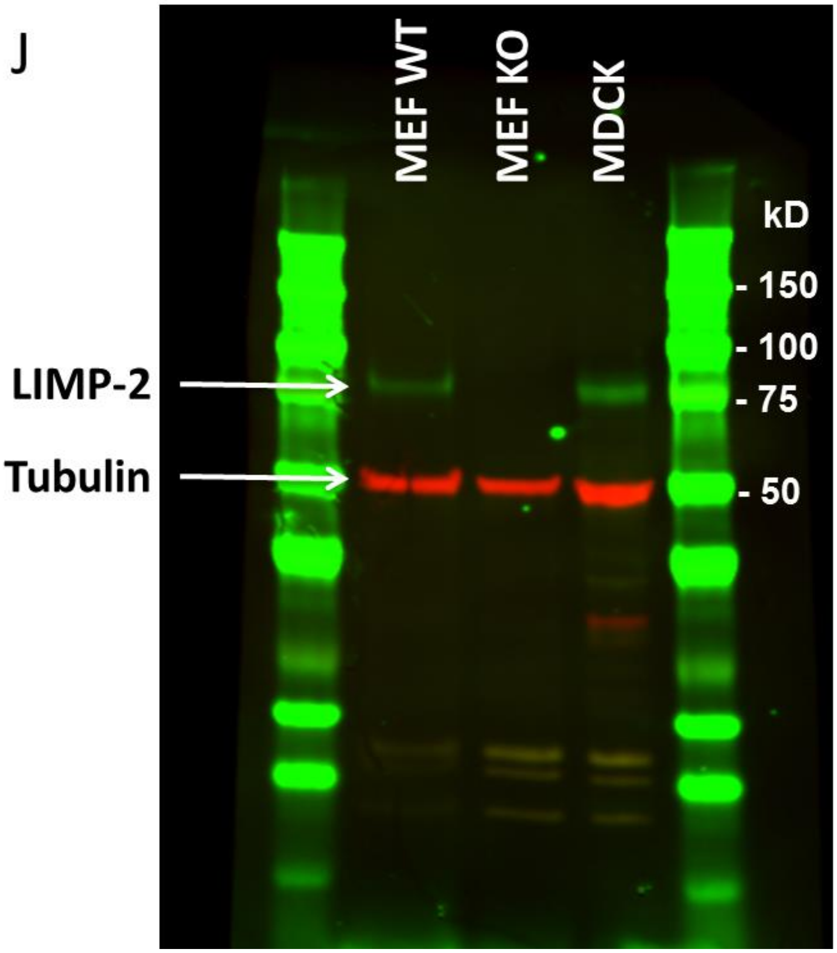

Supplementary Figure 10. Electrophoresis and Immunoblotting of LIMP-2 in cell lysates. A. Multimerization of heterologously expressed LIMP-2/endogenous LIMP-2 captured by UV cross-linking. HeLa cells were transfected with a wild-type and a concatemeric LIMP-2 construct, which consists of two repeated full length cDNA sequences of LIMP-2, used to determine the oligomeric state of LIMP-2. Mock transfection (eGFP expression plasmid) served as a transfection control and allowed for the detection of endogenous LIMP-2 cross-link. One day after transfections, cells were labeled overnight with photoactivatable methionine and leucine. The next day, cells were UV cross-linked, harvested and cell lysates prepared for immunoblotting using LIMP-2 specific antibodies (not UV irradiated cells (-) served as controls). Cross-linking of heterologously expressed LIMP-2 indicate multimers, which can be distinguished by their different migration pattern. The LIMP-2 concatemer verifies the size of the LIMP-2 multimers of endogenous and heterologous expressed wild type LIMP-2. To detect multimers of endogenous LIMP-2, double amount of total protein was loaded in lane three and four. The data shown are representative of at least three independent experiments. B. Whole cell lysates from HeLa cells (WT), HeLa overexpressing LIMP2-myc, LIMP-2 KO MEFs and LIMP-2 WT MEFs were separated under non-denaturing electrophoresis conditions, thus preserving the native state of the proteins. Shown at bottom is a section of the gel with darker contrast. 12 µg of protein was loaded in each lane C. Same samples prepared for B were mixed with SDS sample buffer and proteins separated via standard SDS-PAGE. D.

Demonstration of the interaction of two differently tagged LIMP-2 constructs via co-immunoprecipitation. HeLa cells were co-transfected with LIMP-2-myc and LIMP-2-HA constructs or GFP, respectively, for 48 h. The lysate fractions show that the co-expression of the LIMP-2 constructs was successful. LIMP-2-myc was precipitated with an anti-myc antibody. Staining of the membrane with anti-HA antibody revealed co-immunoprecipitation of LIMP-2-HA. In the antibody control the myc-antibody was incubated with beads only. The asterisk denotes an unspecific band observed above the LIMP-2 signal. E. Detection by Western blot of endogenous LIMP-2 dimer in MDCK cells on a Mini-PROTEAN® TGX Stain-Free™ Precast Gel with Tris-Glycine SDS running buffer. From left: molecular weight standard (Bio-Rad Precision Plus Protein™ Dual Color Standard), whole cell lysate (solubilized for 2 h in 2% Dodecyl Maltoside and Cholesterol Hemisuccinate 10:1 w/v solution (Anatrace) after brief vortexing and sonication), and membrane preparations (same solubilization method followed by a 30 min high speed centrifugation to eliminate the insoluble fraction at 39102 x *g*. Western blot of the protein gel on a nitrocellulose membrane was completed using an iBlot blotting system (ThermoFisher Scientific), followed by detection using an iBind Western device (ThermoFisher Scientific). Primary antibody was rabbit anti-LIMP-2 antibody, and secondary antibody was goat-anti-rabbit IgG HRP conjugate. Imaging was completed after SuperSignal™ West Pico Chemiluminescent substrate detection (ThermoFisher Scientific) using a ChemiDoc Imaging System (Bio-Rad). The lower band detected was the LIMP-2 monomer, and the higher protein band was LIMP-2 dimer/oligomer. F, G, H, I, and J: uncropped raw images of Fig. 4C, Supplementary Fig. 10 B, C, and D, and Fig. 5D, respectively, with molecular makers shown.

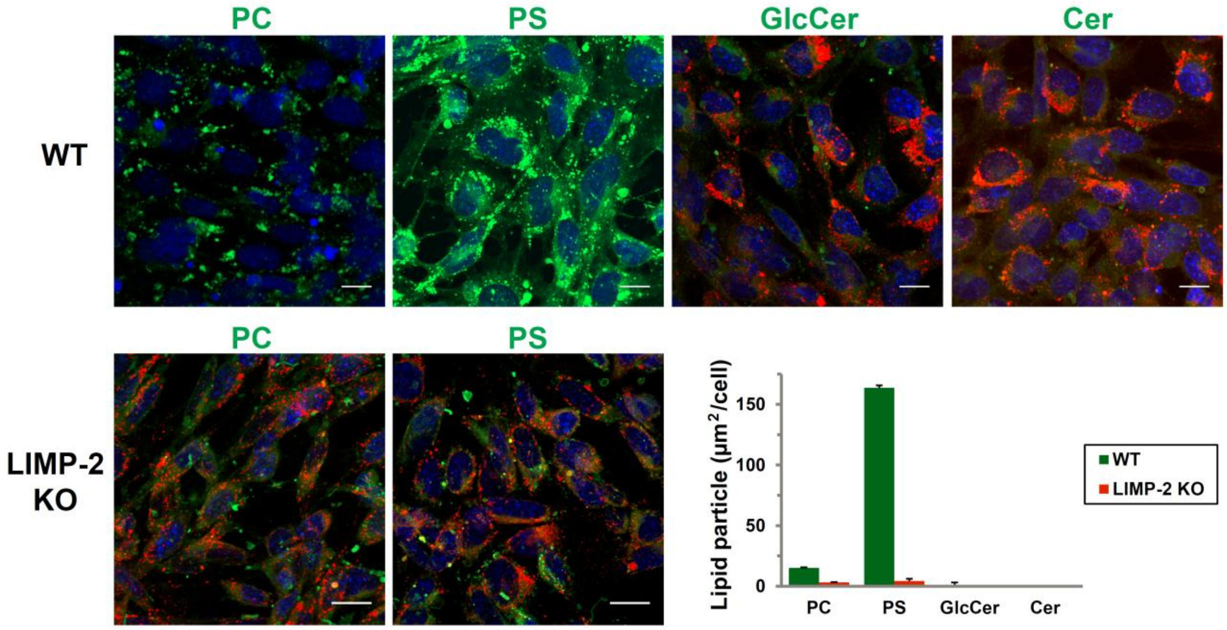

Supplementary Figure 11. PS is the preferred substrate for LIMP-2 mediated uptake of solubilized lipids. Top-Fluor (green) conjugated lipids were first dissolved in BSA. 5 µM of diluted lipids were mixed with lysotracker (red) for 60 min uptake at 37°C. GlcCer, glucosylceramide. Cer, ceramide. Scale bar: 15 µm.

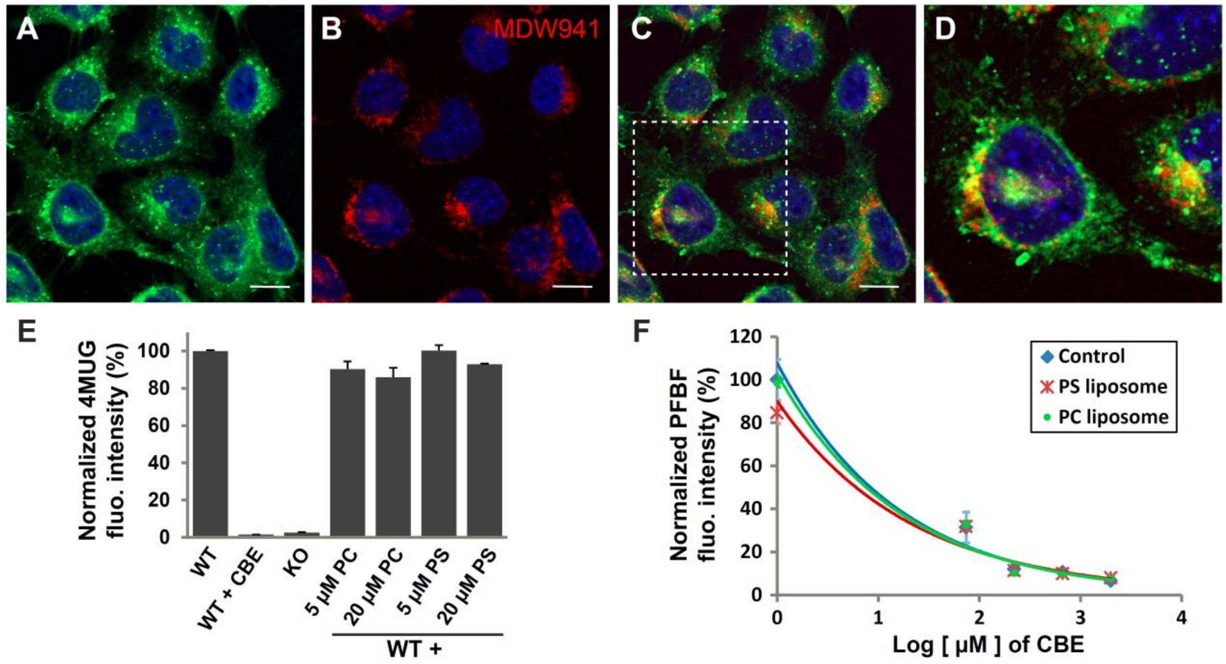

Supplementary Figure 12. LIMP-2 mediated lysosomal delivery of PS liposomes did not interfere with GCase activity. (A-D) Detection of GCase activity with probe MDW941 (red) after 60 min of PS liposome (green) uptake. 23% (N=43) of MDW941-labeled lysosomal GCasewas colocalized with TopFluor PS vesicles. (D) Enlarged view of the square in (C). (E) GCase activity assay with substrate 4MUG. (F) GCase activity assay with substrate PFBF in WT MEF. CBE, GCase inhibitor conduritol B epoxide. Results are expressed as means ± SE. Blue, DAPI. Scale bar: 12 µm.

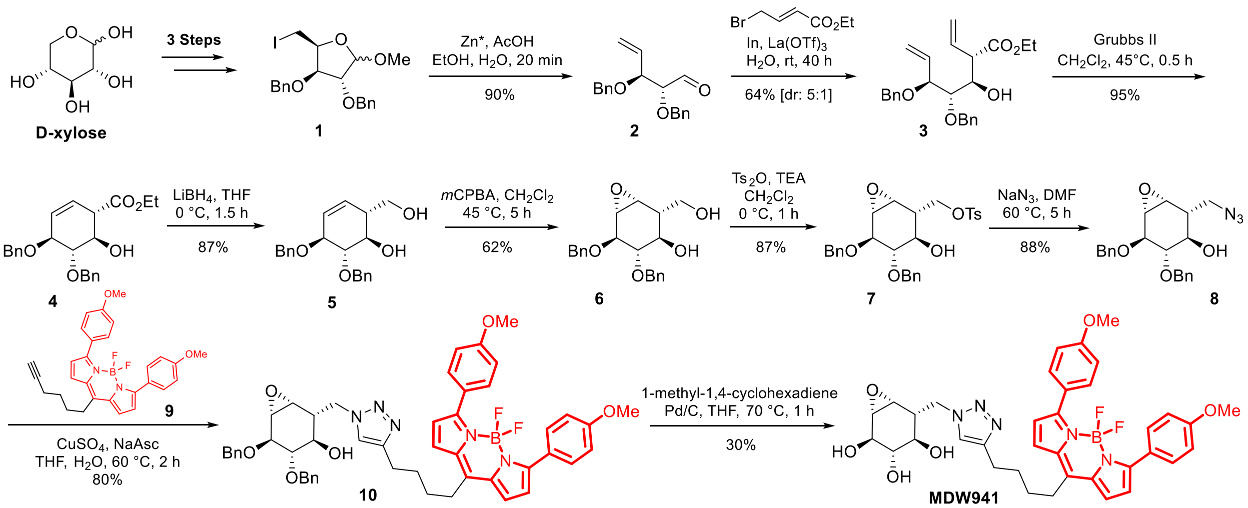

Supplementary Figure 13. Synthesis of MDW941.

Phospholipid	Mixed Acyl Chains	$[M+H]^+$
Phosphatidylcholine	Palmitoyl-Oleoyl (16:0/18:1)	760.58
Phosphatidylcholine	Myristoyl-Oleoyl (14:0/18:1) and Palmitoyl-Palmitoleoyl (16:0/16:1)	732.55
Phosphatidylcholine	Dioleoyl (18:1/18:1)	786.59
Phosphatidylcholine	Palmitoleoyl-Oleoyl (16:1/18:1) and Palmitoyl-Linoleoyl (16:0/18:2)	758.56
Phosphatidylethanolamine	Dioleoyl (18:1/18:1), Stearoyl-Linoleoyl (18:0/18:2) and/or Gadoleoyl-Palmitoleoyl (20:1/16:1)	744.55
Phosphatidylethanolamine	Stearoyl-Oleoyl (18:0/18:1), Arachidoyl-Palmitoleoyl (20:0/16:1) and/or Gadoleoyl-Palmitoyl (20:1/16:0)	746.56

Supplementary Table 1. Summarization of phospholipid species identified in LIMP-2 preparations using LC/MS and MS/MS, listed in order of abundance.

Protein	Liposome	ka (1/Ms)	kd (1/s)	K_D (M)
LIMP-2 Dimer	DOPS(3):DOPC(2)	1.38e3	1.43e-3	9.3(±1.0)e-7
	POPS	2.61e3	2.12e-3	8.6(±0.5)e-7

Supplementary Table 2. SPR parameters of LIMP-2 dimer binding to DOPS(3)DOPC(2) and POPS liposomes at pH 7.5, using global kinetic fitting models of single species binding to single binding site. The apparent K_D (K_d/K_a) values were calculated from a global 1:1 kinetic fitting model for all concentrations used in duplicated experiments. Immobilized levels of DOPS:DOPC (3:2) and POPS on the L1 SPR chip were 8900, 7500 and 6000 units, respectively. The weak LIMP-2 monomer binding parameters cannot be determined meaningfully.

constructs	primers
C-terminal BAP-tagged LIMP-2<br><br>and GCase	R: 5'-TTAATTGCGGCCGCTCATCATTCGTGCCATTCGATTTTCTGAGCTTCGGAAATA TCGTTCAGGCCTCCGTGATGGTGGTGATGATGGGTGTTGATCAT-3';<br>F: 5'-AATTAAGGATCCACCATGGAGACCGACACCCTCCTGCTGTGGGTGCTG-3'. Correction primers: F: 5'-tcacggaggcctgaacgatattttcgaagctcagaa; R: 5'-ttctgagcttcgaaaatatcgttcaggcctccgtga
LIMP-2 F151D	F: 5'-CCAGGTGCATgacCTGAGGGAGA-3'; R: 5'-CTCCACTCGATCACGGTC-3'
LIMP-2 H150A (on F151D)	F: GAGCCAGGTGgcaGACCTGAGGGAG; R: 5'-CACTCGATCACGGTCAGC-3'
LIMP-2 H150AF151D	F: 5'-GTCCCAGGTGgccgacCTGCGCGAGATC; R: 5'-CACTCGATCACGGTCAGC
LIMP-2-A299-HA	5'-GGTATAAAGTTCCTGCAGGATCATACCCATACGATGTTCCAGATTAC GCTGGTTCT CTGCAG AAATATTAGCCA-3' and its complementary strand
LIMP-2-VN155_Myc_Rev	5'-GCTCCTCGAGTCACAGATCCTCTTCTGAGATGAGTTTTTGTTCGGCGGTGAGATAGACG
LIMP-2-VN_For	5'-GGAGGTGGCGGGGAATCCGGAGGAGGGATGGTGAGCAAGGGCGAGG-3'
LIMP-2-VC_For	5'-GGAGGTGGCGGGGAATCCGGAGGAGGGGACAAGCAGAAGAACGGCATC-3'
LIMP-2-VC155_HA_Rev	5'-GCTCCTCGAGTTAAGCGTAATCTGGAACATCGTATGGGTACTTGTACAGCTCGTCCATG

Supplementary Table 3. DNA primers used in the studies.

Supplementary Methods

Scheme 1:

The synthesis was modified from the approaches described by Madsen and Overkleeft in their syntheses of cyclophellitol1 and MDW9412, respectively. The route was optimized for a single compound (MDW941), resulting in a shortened synthetic sequence (14→12 steps, longest linear sequence), achieved by circumventing a protecting group exchange as described in the initial disclosure.2 Further optimizations to the experimental procedures resulted in enhanced material throughput.

General Methods: All commercially available chemicals, reagents and solvents were used as received. Reactions were monitored by thin layer chromatography (TLC) performed on Analtech, Inc. silica gel GF 250 µm plates and were visualized with ultraviolet (UV) light (254 nm) and/or $KMnO_4$ staining or by UPLC-MS (Waters Acquity, ESCI (ESI +/-, APCI +/-)). Gas chromatography – mass spectrometry (GC-MS) was performed with an Agilent 5890 GC Oven and an Agilent 5973 Mass Selective Detector. Silica gel flash chromatography was performed with RediSep®Rf normal phase silica flash columns on a CombiFlash Rf system from Teledyne Isco, Inc. Nuclear magnetic resonance (NMR) spectra chemical shifts are reported in ppm relative to chloroform (^{1}H, δ = 7.26 ppm and ^{13}C, δ = 77.0 ppm) or methanol (^{1}H, δ = 3.31 ppm and ^{13}C, δ = 49.0 ppm). The NMR peak multiplicities are denoted as follows: s, singlet; d, doublet; t, triplet; q, quartet; m, multiplet; br s, broad singlet. High-resolution mass spectra (HRMS) were acquired on an Agilent model 6220 MS(TOF).

Compound **2**:

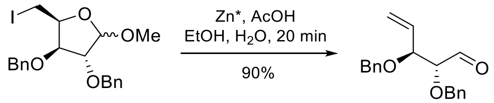

1 2

(2*R*,3*S*)-2,3-bis(benzyloxy)pent-4-enal (2): To a round bottom flask containing Zn dust (4.0 g, 61 mmol) was added 1 N HCl (30 mL) and the mixture was stirred at room temperature for 30 minutes. The HCl was removed by decanting and the resulting activated Zn dust was washed with EtOH (3x) and heptane (2x). The solid was placed under vacuum for 30 minutes and gently heated with a heat gun. To a separate round bottom flask charged with iodide **1** (3.0 g, 6.6 mmol) was added EtOH (60 mL), water (3 mL) and acetic acid (1.5 mL) followed by the activated Zn dust in one portion. The reaction mixture was stirred at room temperature for 20 minutes after which TLC indicated consumption of starting material iodide **1**. EtOAc was added and the mixture was washed with saturated aq. $NaHCO_3$ (2x), water (2x) and brine. The organic phase was dried with anhydrous $MgSO_4$ and concentrated under reduced pressure. The resulting crude colorless oil was purified by silica gel chromatography (0→50%, EtOAc in Heptane) to afford **2** (1.76 g, 90% yield) as a colorless oil. Note: aldehyde **2** degrades upon storage and should be used immediately after purification. ^{1}H NMR (400 MHz, $CDCl_3$) δ 9.69 (s, 1H), 7.41 - 7.20 (m, 10H), 5.94 (ddd, *J* = 7.6, 10.1, 17.4 Hz, 1H), 5.41 - 5.31 (m, 2H), 4.76 (d, *J* = 11.7 Hz, 1H), 4.65 (d, *J* = 12.1 Hz, 1H), 4.63 (d, *J* = 12.1 Hz, 1H), 4.37 (d, *J* = 12.1 Hz, 1H), 4.17 (dd, *J* = 4.1, 7.6 Hz, 1H), 3.84 (d, *J* = 3.9 Hz, 1H). The spectroscopic data was consistent with that reported previously by Madsen et al.[1]

Compound **3**:

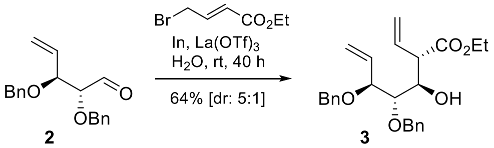

2 3

ethyl (2*S*,3*R*,4*S*,5*S*)-4,5-bis(benzyloxy)-3-hydroxy-2-vinylhept-6-enoate (3): To a 200 mL round bottom flask containing aldehyde **2** (1.7 g, 5.7 mmol) was added deionized water (40 mL) followed by ethyl bromocrotonate (2.77 mL, 20.1 mmol, 3.5 equiv.), lanthanum(III) trifluoromethanesulfonate (6.93 g, 11.5 mmol, 2 equiv.) and indium powder (1.98 g, 17.2 mmol, 3 equiv., 325 mesh). The reaction mixture was stirred vigorously at room temperature for 40 hours until LCMS indicated only a small amount of starting material aldehyde **2** remaining. The mixture was filtered through a plug of Celite, and the solids were rinsed with EtOAc. Brine was added to the filtrate, and the resultant mixture was extracted with EtOAc (2x). The combined organic phases were dried with anhydrous $MgSO_4$ and concentrated under reduced pressure. The crude product was purified by silica gel chromatography (15→50%, EtOAc in Heptane) to afford **3** (1.50 g, 64% yield) as a ~5:1 mixture of diasteromers, favoring the desired, as a colorless oil. The minor diastereomer is purged in subsequent steps. ^{1}H NMR (400 MHz, $CDCl_3$) δ 7.43 - 7.26 (m, 10H), 5.82 (ddd, *J* = 8.0, 10.0, 17.5 Hz, 1H), 5.71 (td, *J* = 9.8, 17.2 Hz, 1H), 5.45 - 5.35 (m, 2H), 5.17 (d, *J* = 9.8 Hz, 1H), 5.06 (d, *J* = 17.2 Hz, 1H), 5.00 (d, *J* = 11.3 Hz, 1H), 4.61 (dd, *J* = 11.5, 14.6 Hz, 2H), 4.41 (d, *J* = 11.7 Hz, 1H), 4.19 (t, *J* = 7.6 Hz, 1H), 4.11 (q, *J* = 7.0 Hz, 2H), 3.98 (d, *J* = 9.0 Hz, 1H), 3.54 (d, *J* = 7.8 Hz, 1H), 3.28 (t, *J* = 9.2 Hz, 1H), 2.37 (br. s., 1H), 1.22 (t, *J* = 7.2 Hz, 3H). The spectroscopic data was consistent with that reported previously by Madsen et al.[1]

Compound **4**:

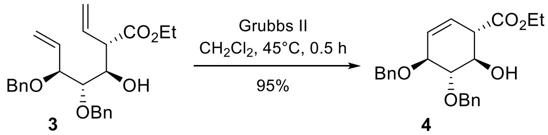

3 4

ethyl (1*S*,4*S*,5*S*,6*R*)-4,5-bis(benzyloxy)-6-hydroxycyclohex-2-ene-1-carboxylate (4): To a round bottom flask containing **3** (1.4 g, 3.4 mmol) was added CH_2Cl_2 (80 mL) followed by Grubbs second generation catalyst (290 mg, 0.34 mmol, 0.1 equiv.). The round bottom was equipped with a Vigreux column and the mixture was heated to reflux (45 °C) for 30 minutes, whereupon TLC indicated consumption of starting material **3**. The mixture was concentrated under reduced pressure and purified by silica gel chromatography (15→50%, EtOAc in Heptane) to afford **4** (1.24 g, 95% yield) as a light beige oil. ^{1}H NMR (400 MHz, $CDCl_3$) δ 7.38 - 7.27 (m, 10H), 5.80 (td, *J* = 2.3, 10.1 Hz, 1H), 5.70 - 5.65 (m, 1H), 4.96 (d, *J* = 11.7 Hz, 1H), 4.80 (d, *J* = 11.3 Hz, 1H), 4.76 - 4.63 (m, 2H), 4.27 - 4.11 (m, 4H), 3.65 (dd, *J* = 7.4, 9.8 Hz, 1H), 3.30 - 3.20 (m, 1H), 2.92 (br. s., 1H), 1.28 (t, *J* = 7.2 Hz, 3H). UPLC-MS (ESI) calculated for $C_{23}H_{26}O_5Na$ $[M+Na]^+$ 405.2, found 405.3. The spectroscopic data was consistent with that reported previously by Madsen et al.[1]

Compound **5**:

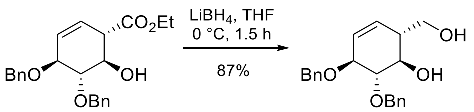

4 5

(1*R*,2*R*,5*S*,6*S*)-5,6-bis(benzyloxy)-2-(hydroxymethyl)cyclohex-3-en-1-ol (5): To a round bottom flask containing ester **4** (1.03 g, 2.68 mmol) was added THF (30 mL) and the mixture was cooled to 0 °C. Next, lithium borohydride

(4.0 mL, 8.0 mmol, 3 equiv., 2M in THF) was added over a period of 5 minutes, and the reaction mixture was stirred at 0 °C for 1 hour 30 minutes after which TLC indicated consumption of starting material ester **4**. The reaction was carefully quenched by the slow addition of 0.5 M $KHSO_4$ and slowly warmed to room temperature. The mixture was extracted with CH_2Cl_2 (3x) and the combined organic phases were washed with brine, dried with anhydrous $MgSO_4$ and concentrated under reduced pressure. The crude product was purified by silica gel chromatography (20→60%, EtOAc in Heptane) to afford **5** (795 mg, 87% yield) as an off-white crystalline solid. ^{1}H NMR (400 MHz, $CDCl_3$) δ 7.39 - 7.28 (m, 10H), 5.78 (td, *J* = 2.3, 10.1 Hz, 1H), 5.55 - 5.49 (m, 1H), 5.03 (d, *J* = 11.3 Hz, 1H), 4.73 (d, *J* = 11.3 Hz, 1H), 4.72 (d, *J* = 11.3 Hz, 1H), 4.64 (d, *J* = 11.3 Hz, 1H), 4.23 - 4.17 (m, 1H), 3.82 - 3.75 (m, 1H), 3.74 - 3.62 (m, 3H), 2.56 - 2.47 (m, 1H), 2.27 (br. s., 2H). UPLC-MS (ESI) calculated for $C_{21}H_{24}O_4Na$ $[M+Na]^+$ 363.2, found 363.2. The spectroscopic data was consistent with that reported previously by Madsen et al.[1]

Compound **6**:

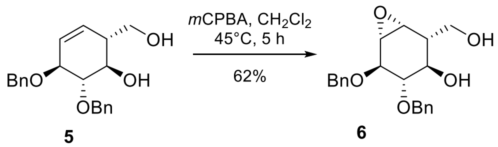

(1*R*,2*R*,3*R*,4*S*,5*R*,6*R*)-4,5-bis(benzyloxy)-2-(hydroxymethyl)-7-oxabicyclo[4.1.0]heptan-3-ol (6): To a round bottom flask containing olefin **5** (749 mg, 2.20 mmol) was added CH_2Cl_2 (10 mL) followed by *m*CPBA (740 mg, 3.3 mmol, 1.5 equiv., 77% purity) in one portion. The round bottom was equipped with a Vigreux column and the mixture was heated to reflux (45 °C) for 5 hours after which TLC indicated consumption of starting material olefin **5**. The reaction mixture was concentrated under reduced pressure and then purified directly by silica gel chromatography (0→100%, EtOAc in Heptane) to afford **6** (485 mg, 62%) as a white solid. ^{1}H NMR (400 MHz, $CDCl_3$) δ 7.44 - 7.26 (m, 12H), 4.96 (d, *J* = 11.3 Hz, 1H), 4.82 (d, *J* = 11.3 Hz, 1H), 4.74 - 4.61 (m, 2H), 4.07 - 3.98 (m, 1H), 3.95 - 3.90 (m, 1H), 3.82 (d, *J* = 7.8 Hz, 1H), 3.54 - 3.45 (m, 1H), 3.45 - 3.36 (m, 1H), 3.28 (d, *J* = 2.3 Hz, 1H), 3.16 (d, *J* = 3.5 Hz, 1H), 2.24 - 2.12 (m, 1H). UPLC-MS (ESI) calculated for $C_{21}H_{24}O_5Na$ $[M+Na]^+$ 379.2, found 379.1. The spectroscopic data was consistent with that reported previously by Madsen et al.[1]

Compound **7**:

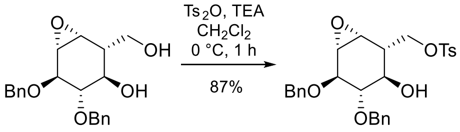

((1*R*,2*R*,3*R*,4*S*,5*R*,6*R*)-4,5-bis(benzyloxy)-3-hydroxy-7-oxabicyclo[4.1.0]heptan-2-yl)methyl 4-methylbenzenesulfonate (7): To a round bottom flask containing alcohol **S6** (228 mg, 0.640 mmol) was added CH_2Cl_2 (5 mL) and the mixture was cooled to 0 °C. *p*-Toluenesulfonic anhydride (230 mg, 0.70 mmol, 1.1 equiv.) in CH_2Cl_2 (3 mL) was added over a period of 2 minutes and then triethylamine (133 µL, 0.960 mmol, 1.5 equiv.) was added over a period of 5 minutes. The reaction was stirred at 0 °C for 1 hour after which TLC indicated consumption of alcohol **6**. The reaction mixture was quenched by the addition of 0.5 M $KHSO_4$ (5 mL). The mixture was extracted with CH_2Cl_2 (3x) and the combined organic phases were dried with anhydrous $MgSO_4$ and concentrated under reduced pressure. The crude amber residue was purified by silica gel chromatography (30→100%, EtOAc in Heptane) to afford **7** (283 mg, 87% yield) as a colorless gum. ^{1}H NMR (400 MHz, $CDCl_3$) δ 7.81

(d, J = 8.2 Hz, 2H), 7.39 - 7.23 (m, 12H), 4.92 (d, J = 11.3 Hz, 1H), 4.80 (d, J = 11.3 Hz, 1H), 4.66 (d, J = 11.3 Hz, 1H), 4.61 (d, J = 11.3 Hz, 1H), 4.45 (dd, J = 3.9, 9.8 Hz, 1H), 4.17 - 4.09 (m, 1H), 3.78 (d, J = 8.2 Hz, 1H), 3.38 - 3.27 (m, 2H), 3.22 - 3.14 (m, 2H), 2.44 (s, 3H), 2.41 (s, 1H), 2.30 (dt, J = 2.9, 9.3 Hz, 1H). UPLC-MS (ESI) calculated for $C_{56}H_{61}O_{14}S_2$ $[2M+H]^+$ 1021.4, found 1022.1.

Compound **8**:

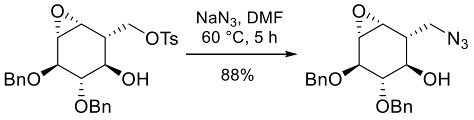

7 **8**

(1*R*,2*R*,3*R*,4*S*,5*R*,6*R*)-2-(azidomethyl)-4,5-bis(benzyloxy)-7-oxabicyclo[4.1.0]heptan-3-ol (8): To a round bottom flask containing tosylate **7** (275 mg, 0.539 mmol) in DMF (3 mL) was added sodium azide (177 mg, 2.7 mmol, 5 equiv.) in one portion. The reaction mixture was heated to 40 °C for 1 hour, whereupon the temperature was increased to 60 °C and heated for 4 hours. The reaction mixture was cooled to room temperature, poured into water and extracted with Et_2O (3x). The combined organic phases were washed with saturated aq. $NaHCO_3$ (2x) and brine (2x). The organic phase was dried with $MgSO_4$ and concentrated under reduced pressure to afford **8** (180mg, 88% yield) as an off-white solid. The crude product was used directly in the subsequent reaction without additional purification. ^{1}H NMR (400 MHz, $CDCl_3$) δ 7.42 - 7.28 (m, 10H), 4.96 (d, J = 11.3 Hz, 1H), 4.83 (d, J = 11.3 Hz, 1H), 4.69 (d, J = 11.3 Hz, 1H), 4.65 (d, J = 11.3 Hz, 1H), 3.88 - 3.80 (m, 2H), 3.50 (dd, J = 9.4, 12.1 Hz, 1H), 3.39 - 3.32 (m, 2H), 3.32 - 3.24 (m, 1H), 3.23 (d, J = 3.5 Hz, 1H), 2.25 - 2.00 (m, 2H). ^{13}C NMR (100 MHz, $CDCl_3$) δ 138.05, 137.27, 128.61, 128.58, 127.99, 127.88, 83.37, 79.26, 74.85, 72.58, 66.61, 54.52, 53.92, 51.04, 41.85. HRMS (ESI) calculated for $C_{21}H_{23}N_3NaO_4$ $[M+Na]^+$ 404.1581, found 404.1577.

Compound **10**:

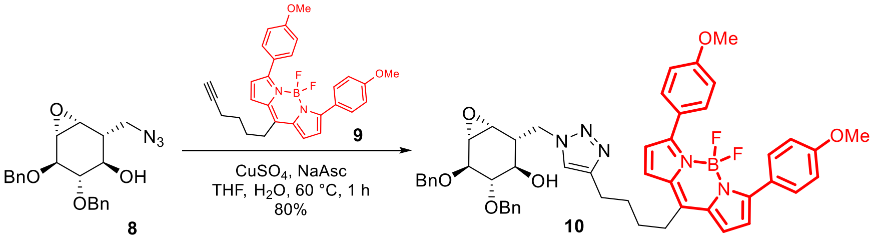

8 **10**

(1*R*,2*R*,3*R*,4*S*,5*R*,6*R*)-4,5-bis(benzyloxy)-2-((4-(4-(5,5-difluoro-3,7-bis(4-methoxyphenyl)-5*H*-4λ^4,5 λ^4-dipyrrolo[1,2-*c*:2',1'-*f*][1,3,2]diazaborinin-10-yl)butyl)-1*H*-1,2,3-triazol-1-yl)methyl)-7-oxabicyclo[4.1.0]heptan-3-ol (10): To a 1-dram vial containing azide **8** (40 mg, 0.10 mmol) and alkyne **9**[2] (66 mg, 0.14 mmol, 1.3 equiv.) was add THF (1.5 mL) and water (5 drops) followed by $CuSO_4$ (16 mg, 0.10 mmol, 1 equiv.) and sodium ascorbate (20 mg, 0.10 mmol, 1 equiv.) and the mixture was heated to 45 °C for 1 hour. The reaction mixture was cooled to room temperature and purified directly by silica gel chromatography (0→10%, MeOH in CH_2Cl_2) to afford **10** (73 mg, 80% yield) as a dark purple solid. ^{1}H NMR (400 MHz, $CDCl_3$) δ 7.83 (d, J = 8.6 Hz, 4H), 7.39 - 7.30 (m, 8H), 7.30 - 7.21 (m, 5H), 6.92 (d, J = 9.0 Hz, 4H), 6.59 (d, J = 3.9 Hz, 2H), 4.93 (d, J = 11.3 Hz, 1H), 4.82 - 4.73 (m, 2H), 4.63 (d, J = 11.7 Hz, 2H), 4.53 (dd, J = 8.8, 13.9 Hz, 1H), 3.83 (s, 6H), 3.78 (d, J = 7.4 Hz, 1H), 3.39 - 3.26 (m, 2H), 3.14 (d, J = 3.5 Hz, 1H), 3.07 (m, 1H), 3.01 - 2.90 (m, 2H), 2.85 - 2.75 (m, 2H), 2.59 (br. s., 1H), 2.52 - 2.43 (m, 1H), 1.94 - 1.81 (m, 4H). ^{13}C NMR (100 MHz, $CDCl_3$) δ 160.50, 157.48, 147.06, 144.75, 137.99, 137.19, 136.07, 130.93 (t, J = 4.4 Hz), 128.52, 128.50, 128.02, 127.91, 127.83, 126.78, 125.14, 119.94, 113.67, 83.13, 78.97, 74.82, 72.51, 66.63, 55.18,

54.04, 53.81, 49.66, 42.60, 32.86, 30.28, 29.35, 24.90. HRMS (ESI) calculated for $C_{50}H_{51}BF_2N_5O_6$ $[M+H]^+$ 866.3903, found 866.3901.

Compound **MDW941**:

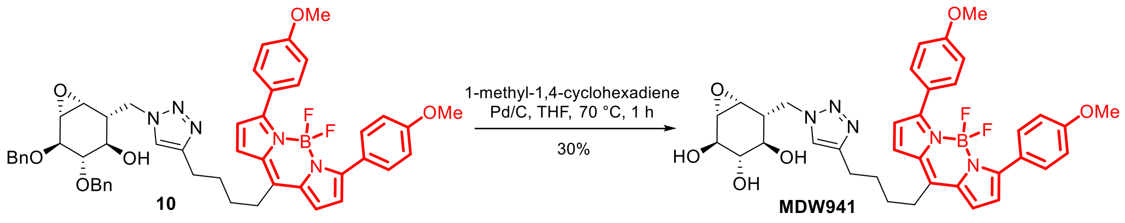

(1*S*,2*R*,3*S*,4*R*,5*R*,6*R*)-5-((4-(4-(5,5-difluoro-3,7-bis(4-methoxyphenyl)-5*H*-4λ[4],5 λ[4]-dipyrrolo[1,2-*c*:2',1'-*f*][1,3,2]diazaborinin-10-yl)butyl)-1*H*-1,2,3-triazol-1-yl)methyl)-7-oxabicyclo[4.1.0]heptane-2,3,4-triol (**MDW941**): To a 2-dram vial containing **10** (34 mg, 0.039 mmol) was added THF (1.5 mL) followed by Pd/C (50 mg, 0.047 mmol, 1.2 equiv., 10% wt.). The reaction was heated to 70 °C and methyl-1,4-cyclohexadiene (0.44 mL, 3.9 mmol, 100 equiv.) was added in portions over a period of 30 minutes. The reaction was heated an additional 30 minutes at 70 °C after which LCMS indicated consumption of starting material. The reaction mixture was cooled to room temperature and filtered through a 0.2 micron Acrodisc syringe filter, washing with CH_2Cl_2 and the resulting solution was concentrated under reduced pressure. The crude product was purified by silica gel chromatography (0→10%, MeOH in CH_2Cl_2) to afford **MDW941** (9 mg, 30% yield) as a dark purple solid. ^{1}H NMR (400 MHz, CD_3OD) δ 7.84 (d, *J* = 8.6 Hz, 4H), 7.77 (s, 1H), 7.44 (d, *J* = 4.3 Hz, 2H), 6.97 (d, *J* = 8.6 Hz, 4H), 6.69 (d, *J* = 4.3 Hz, 2H), 4.78 (dd, *J* = 3.9, 14.0 Hz, 1H), 4.65 - 4.54 (m, 1H), 3.85 (s, 6H), 3.60 (d, *J* = 7.8 Hz, 1H), 3.25 - 3.18 (m, 1H), 3.18 - 3.10 (m, 1H), 3.06 (t, *J* = 6.6 Hz, 2H), 3.00 (s, 2H), 2.84 - 2.77 (m, 2H), 2.36 (dt, *J* = 3.9, 9.0 Hz, 1H), 1.92 - 1.77 (m, 4H). ^{13}C NMR (100 MHz, CD_3OD) δ 162.31, 158.93, 148.83, 146.94, 132.36 (t, *J* = 4.4 Hz), 132.31, 128.59, 126.67, 124.50, 121.20, 114.78, 78.41, 72.67, 68.79, 57.65, 55.97, 55.63, 50.84, 44.78, 34.34, 31.17, 30.59, 25.94. HRMS (ESI) calculated for $C_{36}H_{39}BF_2N_5O_6$ $[M+H]^+$ 686.2962, found 686.2957.

Supplementary References:

1. Hansen, F. G., Bundgaard, E. & Madsen, R. A short synthesis of (+)-cyclophellitol. *J. Org. Chem*. 70, 10139 (2005)
2. Witte M.D., Kallemeijn W.W., Aten J., Li K.Y., Strijland A., Donker-Koopman W.E., van den Nieuwendijk A.M.C.H., Bleijlevens B., Kramer G., Florea B.I., Hooibrink B., Hollak C.E.M., Ottenhoff R., Boot R.G., van der Marel G.A., Overkleeft H.S. & Aerts J.M.F.G. Ultra-sensitive in situ visualization of active glucocerebrosidase molecules. *Nat. Chem. Biol.* 6, 907 (2010)
