# Supplementary material for: Lysosomal integral membrane protein-2 as a phospholipid receptor revealed by biophysical and cellular studies
Source: Nat Commun. 2017 Dec 4;8:1908. doi: 10.1038/s41467-017-02044-8 (PMC5712522; doi:10.1038/s41467-017-02044-8)
Supplement: Supplementary file 3 — Description of Additional Supplementary Files [file 41467_2017_2044_MOESM3_ESM.pdf]

Description of Additional Supplementary Files

File Name: Supplementary Movie 1
Description: Overview of the electrostatic potential surface of the LIMP-2 dimer.

File Name: Supplementary Movie 2
Description: Imaging of LIMP-2 ligand binding sites and dimerization interface. The subunits of lipid bound dimer are shown as green and cyan ribbons, with the apo LIMP-2 monomer (gray ribbons) superimposed to the cyan subunit of the dimer to show conformational differences. Key peptides are labelled.

File Name: Supplementary Movie 3
Description: Time-lapse imaging of LIMP-2 trafficking in a MDCK cell.
